# The SOX17/miR-371-5p/SOX2 axis inhibits EMT, stem cell properties and metastasis in colorectal cancer

**DOI:** 10.18632/oncotarget.3603

**Published:** 2015-03-15

**Authors:** Yuling Li, Zhenbing Lv, Guoyang He, Jianmei Wang, Xiaojing Zhang, Guifeng Lu, Xiaoli Ren, Feifei Wang, Xiaohui Zhu, Yi Ding, Wenting Liao, Yanqing Ding, Li Liang

**Affiliations:** ^1^ Department of Pathology, Nanfang Hospital, Southern Medical University, Guangzhou, Guangdong Province, People's Republic of China; ^2^ Guangdong Province Key Laboratory of Molecular Tumor Pathology, Guangzhou, Guangdong Province, People's Republic of China; ^3^ Department of General Surgery Two, Nanchong Central Hospital, Nanchong city, Sichuan Province, People's Republic of China; ^4^ The Second Clinical School of Northern Sichuan Medical College, Nanchong city, Sichuan Province, People's Republic of China; ^5^ Department of Pathology, Shenzhen University, Shenzhen, People's Republic of China; ^6^ Department of Radiotherapy, Nanfang Hospital, Southern Medical University, Guangzhou, Guangdong Province, People's Republic of China

**Keywords:** miR-371-5p, SOX17, SOX2, metastasis, colorectal cancer

## Abstract

Cancer stem cells (CSCs) and EMT-type cells, which share molecular characteristics with CSCs, have been believed to play critical roles in tumor metastasis. Although much progress has been garnered in elucidating the molecular pathways that trigger EMT, stemness and metastasis, a number of key mechanistic gaps remain elusive. In the study, miR-371-5p was obviously down-regulated in primary CRC tissues compared with matched adjacent normal mucosa and correlated significantly with differentiation, tumor size, lymphatic and liver metastases. MiR-371-5p could attenuate proliferation, invasion *in vitro* and metastasis *in vivo* in CRC cells. It also suppressed EMT by regulating *Wnt*/β-catenin signaling and strongly decreased the CRC stemness phenotypes. Moreover, demethylation of SOX17 induced miR-371-5p expression and consequently suppressed its direct target SOX2 in CRC cells. MiR-371-5p was necessary for SOX17 mediated cancer-related traits and SOX2 was a functional target of miR-371-5p. A positive relationship between SOX17 and miR-371-5p expression and a negative one between miR-371-5p and SOX2 expression were observed in CRC cell lines and tissues. In conclusion, we identified miR-371-5p as an important “oncosuppressor” in CRC progression and elucidated a novel mechanism of the SOX17/miR-371-5p/SOX2 axis in the regulation of EMT, stemness and metastasis, which may be a potential therapeutic target.

## INTRODUCTION

Colorectal cancer (CRC) is the third most common cancer worldwide and the fourth most common cause of death in China. The high mortality of CRC could, in part, be due to high propensity for recurrence and metastasis [1]. In addition to remodeling the microenvironment to facilitate metastasis, cancer cells also turn on embryonic morphogenesis regulators to undergo the epithelial-mesenchymal transition (EMT) and turn off differentiation programs [2], allowing cancer cells to gain motility, invasion and acquire stem-like properties [3]. Recently, cancer stem cells (CSCs) and EMT-type cells, which share molecular characteristics with CSCs, have been believed to play critical roles in tumor metastasis [4]. CSCs and the EMT process can drive metastatic tumor formation in breast cancer [5]. However, molecular knowledge of metastasis in relation to CSCs and EMT in CRC remains unclear.

Growing evidence indicates that microRNAs (miRNAs) are aberrantly expressed in many human cancers and involve in the initiation, development and metastasis of cancers [6, 7]. Recent findings have noted the interconnections between miRNAs and metastasis, EMT, CSCs [8, 9]. MiR-200c is the predominant member of the miR-200 family, which suppresses EMT [10]. Moreover, a miR-200c-*SOX2*-negative feedback loop regulates stemness, growth and metastasis in colorectal cancer [11]. Although much progress has been garnered in elucidating the molecular pathways that trigger EMT, stemness and metastasis, a number of key mechanistic gaps remain to be explained.

Recently, the altered expression of miR-371-5p was detected using miRNAs array in Ewing's sarcoma [12], gastric cancer [13], carcinoma in situ cells of the testis [14] or cisplatin-resistant germ cell tumor [15]. MiR-371-5p was found to inhibit *PRPF4B* to facilitate the G1/S transition in hepatocellular cancer [16]. However, there has been no published data about the roles of miR-371-5p in tumor metastasis, EMT and stemness.

In this study, we report the suppressive effects of miR-371-5p on EMT, stemness and metastasis in CRC cells. We provide evidence, for the first time, that miR-371-5p, induced by its upstream transcription factor SOX17, can suppress EMT, stemness and metastasis in CRC by targeting *SOX2*, at least in part through inhibiting Wnt/β-catenin pathway.

## RESULTS

### Down-regulation of miR-371-5p correlates with CRC metastasis

**Figure 1:**
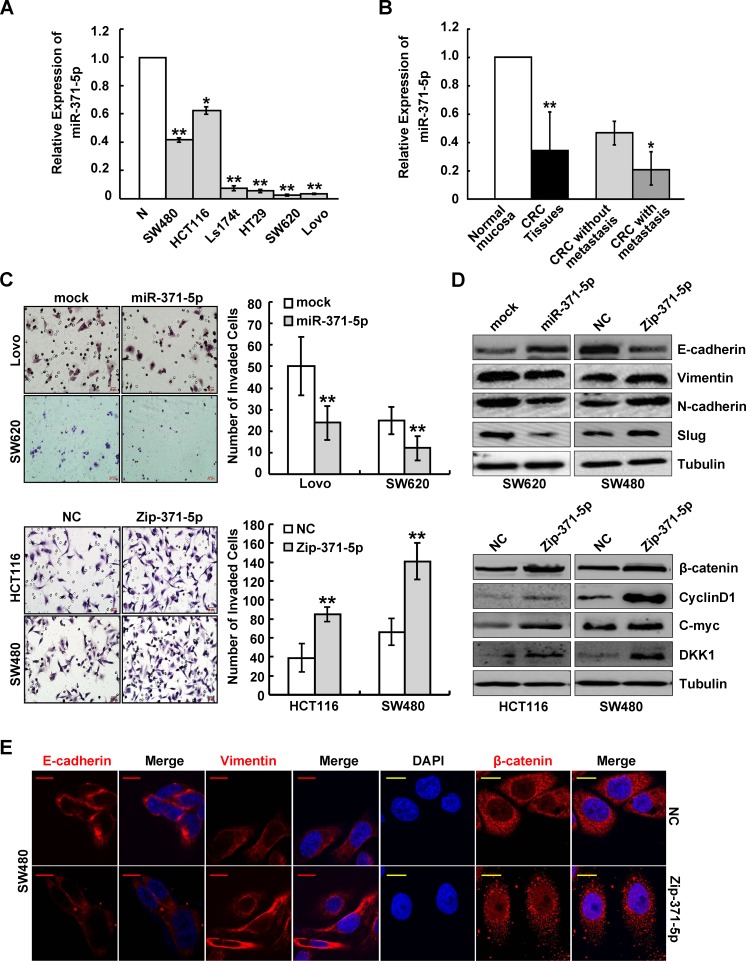
miR-371-5p is associated with CRC metastasis and suppresses invasion and EMT of CRC cells *in vitro* (A) Endogenous expression of miR-371-5p in 6 CRC cell lines and normal colon mucosa (N) by qRT-PCR. The relative expression levels of miR-371-5p in normal colon mucosa were normalized to 1. (B) Expression of miR-371-5p in 100 cases of the primary CRC tissues with or without metastasis and matched adjacent normal mucosa by qRT-PCR. The relative expression levels of miR-371-5p in normal mucosa were normalized to 1. (C) Effect of miR-371-5p ectopic expression or miR-371-5p knockdown on the invasiveness of CRC cells by Boyden chamber. Scale bars represent 20 μm. (D) Expression of EMT related markers and target genes of Wnt/β-catenin signaling in miR-371-5p over-expressing or depleting cells by Western blot. Expression levels were normalized to Tubulin. (E) Immunofluorescence images of E-cadherin, Vimentin expression and nuclear translocation of β-catenin in miR-371-5p depleting cells. Red scale bars represent 10 μm, whereas yellow scale bars represent 5 μm. * P < 0.05, ** P < 0.01. Data represent the mean ± SD.

To investigate the expression pattern and clinicopathologic significance of miR-371-5p in CRC, we first detected miR-371-5p expression in 6 CRC cell lines with different metastatic potentials. Real-time PCR analyses showed that miR-371-5p was obviously down-regulated in 6 CRC cell lines compared with normal colon mucosa. The expression levels of miR-371-5p were lower in high metastatic SW620 and Lovo cell lines than those with low metastatic abilities (Figure 1A). Thus we examined the expression and clinical values of miR-371-5p in 100 cases of paired CRC tissues. The expression of miR-371-5p was obviously lower in primary CRC tissues than in matched adjacent non-tumor mucosa. Strikingly, miR-371-5p was markedly down-regulated in primary CRC tissues with metastasis compared with those without metastasis (Figure 1B). Moreover, miR-371-5p expression correlated significantly with differentiation, tumor size, lymphatic and liver metastases (*p* < 0.05; Supplementary Table 1). Since miR-371-5p and miR-371-3p are derived from a single precursor, we also assessed the expression of miR-371-3p in CRC tissues. However, there was no significant difference of miR-371-3p expression between primary CRC tissues and matched adjacent normal mucosa (Supplementary Figure 1A). The above results suggest a possible link between down-regulation of miR-371-5p and CRC metastasis.

### MiR-371-5p suppresses cell proliferation, invasion and EMT in CRC cells

To explore the potential effects of miR-371-5p, we transduced lentiviral vectors expressing miR-371-5p or repressing miR-371-5p into CRC cells, respectively (Supplementary Figure 1B). Ectopic expression of miR-371-5p inhibited cell proliferation, as shown by MTT and colony formation assays (*p* < 0.05; Supplementary Figure 1C), while knockdown of miR-371-5p enhanced cell proliferation (*p* < 0.05; Supplementary Figure 1D). Over-expression of miR-371-5p reduced the number of invaded CRC cells, while silence of miR-371-5p showed the opposite effect (Figure 1C). We also examined the effect of miR-371-3p inhibitor on proliferation and invasion of CRC cells, and found that miR-371-3p did not affect those properties *in vitro* (*p* > 0.05; Supplementary Figure 2A).

In miR-371-5p depleting cells, a dramatic morphological change was also observed, in which the typical cobblestone-like appearance of cells was replaced by a spindle-like, fibroblastic morphology (Supplementary Figure 2B). In agreement with these observations, we found that miR-371-5p ectopic expression displayed an increased expression of the key epithelial marker E-cadherin, and the down-regulations of the mesenchymal markers N-cadherin, Vimentin and Slug, and vice versa (Figure 1D and 1E, Supplementary Figure 2C). Knockdown of miR-371-5p resulted in the nuclear translocation of β-catenin (Figure 1E), TCF/LEF transcriptional activation (Supplementary Figure 2D) and increased expression of target genes of Wnt/β-catenin signaling including CyclinD1, C-myc and DKK1 (Figure 1D). Taken together, our data suggest that miR-371-5p suppresses cell proliferation, invasion and EMT by regulating β-catenin/TCF activity in CRC.

### MiR-371-5p suppresses stem cell properties and metastasis of CRC cells

**Figure 2:**
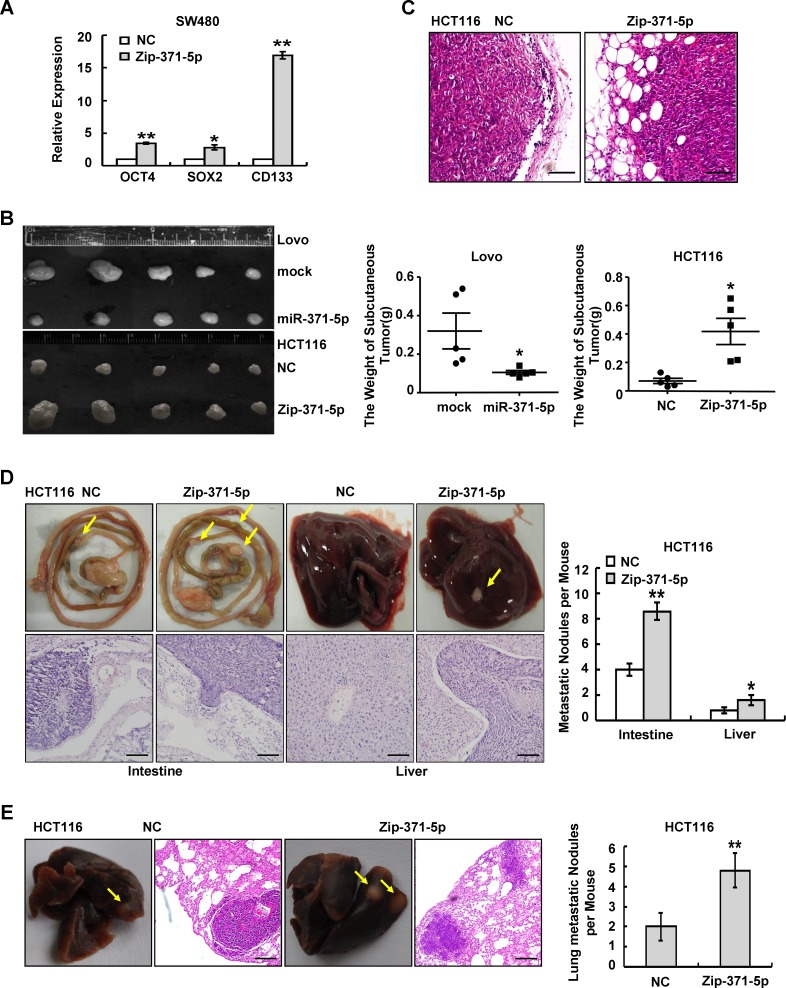
miR-371-5p is sufficient to suppress stem cell properties and metastasis of CRC cells (A) Expression of OCT4, SOX2 and CD133 in SW480 cells treated with Zip-371-5p by qRT-PCR. The relative expression levels in NC cells were normalized to 1. (B) Lovo/mock and Lovo/miR-371-5p cells (1×10^6^), and HCT116/NC and HCT116/Zip-371-5p cells (1×10^6^) were injected in the hindlimbs of nude mice (n = 5). The weight of subcutaneous tumors was measured. (C) Local invasion of subcutaneous tumors in HCT116/NC group or HCT116/Zip-371-5p group by HE staining. Scale bars represent 50 μm. (D) Intestinal and hepatic metastatic nodules after subcutaneous tumors of HCT116/NC and HCT116/Zip-371-5p were transplanted in the mesentery at the tail end of cecum (n = 5) for six weeks. Yellow arrows in top panels point at metastatic nodules. Scale bars in bottom panels represent 50 μm. The number of intestinal or hepatic metastatic nodules per mouse was counted under the microscope. (E) HCT116/NC and HCT116/Zip-371-5p cells (2×10^6^) were injected in the tail vein of nude mice (n = 5) for 2 months. Yellow arrows point at lung metastatic nodules. Scale bars represent 100 μm. The number of lung metastatic nodules per mouse was counted under the microscope. * P < 0.05, ** P < 0.01. Data represent the mean ± SD.

The EMT is known to be a central mechanism responsible for invasiveness and metastasis of breast cancer and is also associated with normal and malignant mammary stem cell function [17]. Since the microRNA-371-373 cluster is thought to be involved in stem cell pluripotency [18, 19], we speculated that miR-371-5p could also induce stemness. Substantially, miR-371-5p knockdown resulted in up-regulations of stem cell pluripotency factors *OCT4* and *SOX2* and stem cell marker *CD133* (Figure 2A). Over-expression of miR-371-5p decreased the ability of cells to develop into spheres, and vice versa (Supplementary Figure 2E). Because of its effects on *in vitro* traits associated with high-grade malignancy, we asked whether miR-371-5p could inhibit tumor growth and metastasis *in vivo*. MiR-371-5p over-expressing cells or miR-371-5p silencing cells were injected into the subcutaneous site of mice, respectively. Noticeably, over-expression of miR-371-5p inhibited primary tumor growth, while silence of miR-371-5p showed the opposite effect (Figure 2B). Unexpectedly, control cell primary tumors were well encapsulated and noninvasive, while miR-371-5p depleting tumors displayed evidence of local invasion (Figure 2C). Accordingly, we used orthotopic transplant to evaluate metastasis in nude mice. Orthotopic transplant of miR-371-5p depleting tumors into nude mice gave rise to metastatic nodules similar in size to control tumors, and showed a significant increase in the number of spontaneous intestinal and liver metastases (Figure 2D). Next, we also examined whether miR-371-5p only limits the ability of tumor cells to disseminate from the primary site, or if it also affects the late stages of metastasis, for example, colonization. We inoculated miR-371-5p depleting cells or control cells into the circulation of nude mice through the tail vein. Knockdown of miR-371-5p in HCT116 cells strikingly enhanced their capacity to seed lung metastases (Figure 2E). MiR-371-5p depleting metastatic tumors in intestine, liver or lung tended to infiltrate the adjacent tissues compared to control groups (Figure 2D and 2E). The above results indicate that miR-371-5p inhibited stemness and metastasis of CRC cells.

### SOX17 transcriptionally regulates miR-371-5p

**Figure 3:**
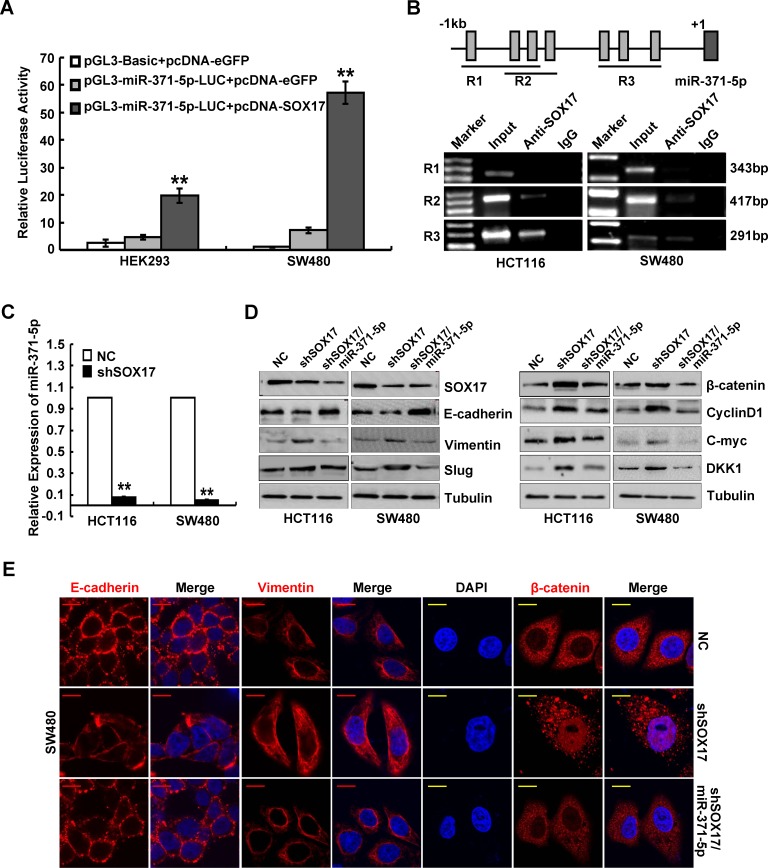
SOX17 transcriptionally regulates miR-371-5p in CRC cells and is sufficient to suppress EMT by regulating miR-371-5p (A) Luciferase activity of miR-371-5p-promoter-luc construct after transfection of SOX17 plasmid in HEK293 and SW480 cells. (B) ChIP assay in HCT116 and SW480 cells. PCR was performed with primers specific for 3 regions in miR-371-5p promoter (R1, R2 and R3), which include 7 putative SOX17 binding sites. Input was used as a positive control, whereas IgG was a negative one. (C) Expression of miR-371-5p in SOX17 depleting HCT116 and SW480 cells by qRT-PCR. The relative expression levels of miR-371-5p in NC cells were normalized to 1. (D) Expression of EMT related markers and target genes of Wnt/β-catenin signaling in cells treated with shSOX17 or shSOX17/miR-371-5p by Western blot. Expression levels were normalized to Tubulin. (E) Immunofluorescence images of E-cadherin, Vimentin expression and nuclear translocation of β-catenin in SW480/NC, SW480/shSOX17 and SW480/shSOX17/miR-371-5p cells. Red scale bars represent 10 μm, whereas yellow scale bars represent 5 μm. * P < 0.05, ** P < 0.01. Data represent the mean ± SD.

To explore whether miR-371-5p expression levels was associated with the promoter hypermethylation, we treated CRC cells with methyltransferase inhibitor 5′AZC or Genistein, and found that 5′AZC or Genistein treatment induced the increased expression of miR-371-5p (Supplementary Figure 3A). However, no CpG islands were found in the 1kb region directly upstream of miR-371-5p (promoter) (Supplementary Figure 3B). We then predicted the possible transcription factor binding sites in the promoter of miR-371-5p by using Consite (http://consite.genereg.net/) and TFsearch (http://www.cbrc.jp/research/db/TFSEARCH.html) databases. The possible binding motifs of SOX17 were found in the promoter of miR-371-5p in both databases. It was observed that *SOX17* effectively stimulated the luciferase activity of miR-371-5p promoter in HEK293 and SW480 cells (Figure 3A). ChIP results also showed that SOX17 could directly bind the region of R2 (−777~−361bp) and R3 (−376~−86bp) in the promoter of miR-371-5p (Figure 3B). Moreover, knockdown of *SOX17* led to decreased expression of miR-371-5p in HCT116 and SW480 cells (Supplementary Figure 3C and Figure 3C). Interestingly, we also found that Genistein treatment in CRC cells induced the increased expression of *SOX17* (Supplementary Figure 3D). In CRC, *SOX17* silence was found to be due to promoter hypermethylation and contribute to aberrant activation of Wnt signaling [20]. Therefore, the above results indicate that demethylation of *SOX17* in CRC can positively regulated miR-371-5p expression.

### *SOX17* is sufficient to suppress metastasis-relevant traits *in vitro* by regulating miR-371-5p

To examine whether miR-371-5p affects the function of SOX17 in the progression of CRC, we stably transduced *SOX17* depleting cells with miR-371-5p and confirmed its over-expression (Supplementary Figure 3C). We found that knockdown of *SOX17* promoted cell proliferation and invasiveness *in vitro*, while reintroduction of miR-371-5p abolished the tumor promoting effects of *SOX17* knockdown on CRC cells (Supplementary Figure 4A). Knockdown of *SOX17* also induced EMT in CRC cells by activating Wnt/β-catenin signaling, as shown by the morphological changes (Supplementary Figure 4B), up-regulations of Vimentin and Slug, down-regulation of E-cadherin (Figure 3D and 3E), nuclear translocation of β-catenin (Figure 3E), TCF/LEF transcriptional activation (Supplementary Figure 4C) and increased expression of target genes CyclinD1, C-myc and DKK1 (Figure 3D). However, miR-371-5p could reverse *SOX17* knockdown-induced EMT (Supplementary Figure 4B and 4C, Figure 3D and 3E). These data make it obvious that miR-371-5p is necessary for SOX17-mediated cell proliferation, invasion and EMT *in vitro*.

### *SOX17* is sufficient to suppress stemness and metastasis *in vivo* by regulating miR-371-5p

**Figure 4:**
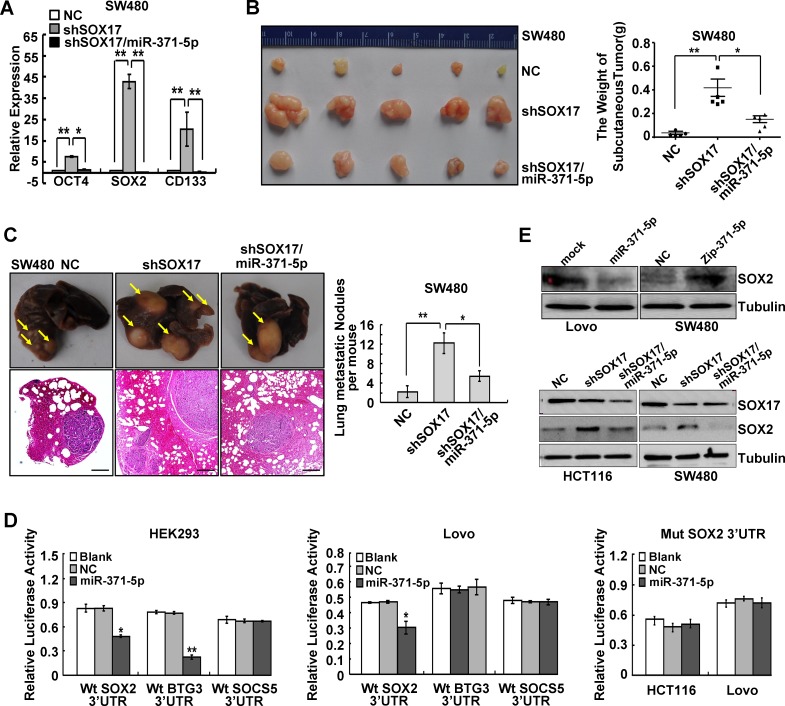
SOX17 is sufficient to suppress stemness and metastasis of CRC cells by regulating miR-371-5p and SOX2 is a direct target of miR-371-5p (A) Expression of OCT4, SOX2 and CD133 in SW480 cells treated with shSOX17 or shSOX17/miR-371-5p by qRT-PCR. The relative expression levels in NC cells were normalized to 1. (B) SW480/NC, SW480/shSOX17 and SW480/shSOX17/miR-371-5p cells (1×10^6^) were injected in the subcutaneous tissue of nude mice (n = 5). The weight of subcutaneous tumors was measured. (C) SW480/NC, SW480/shSOX17 and SW480/shSOX17/miR-371-5p cells (2×10^6^) were injected into the tail vein of nude mice (n = 5) for 2 months. Yellow arrows in top panels point at lung metastatic nodules. Scale bars in bottom panels represent 100 μm. The number of lung metastatic nodules per mouse was counted under the microscope. (D) Luciferase activities of wild-type 3′UTR-SOX2-luc, 3′UTR-BTG3-luc, 3′UTR-SOCS5-luc constructs and mutant 3′UTR-SOX2-luc constructs in cells after transfection of miR-371-5p. (E) SOX2 expression in cells treated with miR-371-5p, Zip-371-5p, shSOX17 or shSOX17/miR-371-5 by Western blot. Expression levels were normalized to Tubulin. * P < 0.05, ** P < 0.01. Data represent the mean ± SD.

We also examined whether miR-371-5p was necessary for SOX17-induced stemness and metastasis of CRC cells. We found that *SOX17* knockdown increased the expression of stem cell pluripotency factors *OCT4*, *SOX2* and stem cell marker *CD133* (Figure 4A) and enhanced sphere-forming capacity of CRC cells (Supplementary Figure 4D), while this promoting effect could be rescued by miR-371-5p (Figure 4A and Supplementary Figure 4D). In the mouse models of tumor growth and metastasis, *SOX17* depleting cells were injected in subcutaneous site of nude mice or into tail vein to seed lung metastases, respectively. Impressively, depletion of *SOX17* enhanced tumor growth (Figure 4B). *SOX17* depleting tumors provided evidence of local invasion compared to control cell primary tumors, while re-introduction of miR-371-5p produced well encapsulated tumors (Supplementary Figure 4E). *SOX17* depleting cells also produced more lung metastases compared to control cells (Figure 4C). However, the constitutive expression of miR-371-5p also rescued *SOX17* knockdown-induced growth and metastasis (Figure 4B and 4C). Based on these results it would be reasonable to conclude that SOX17 is sufficient to inhibit stem cell properties, tumor growth and metastasis *in vivo* by up-regulating miR-371-5p.

### *SOX2* is a direct and functional target of miR-371-5p

To identify effectors of miR-371-5p, we used four algorithms named microRNA.org, TargetScan, miRDB and DIANA-microT-CDS that predict the mRNA targets of a microRNA. Based on the representation of miR-371-5p binding sites in their 3′UTRs, >200 mRNAs were predicted to be regulated by miR-371-5p. Among those mRNAs, *BTG3*, *SOX2* and *SOCS5* were predicted by all four databases. We cloned the 3′UTRs of *BTG3*, *SOX2* and *SOCS5* into a luciferase construct. Reporter assays revealed that miR-371-5p only repressed the luciferase activity of Wt *SOX2* 3′UTR in CRC cells, and it had no effect on the activity of Mut *SOX2* 3′UTR (Supplementary Figure 5A and Figure 4D). Moreover, miR-371-5p ectopic expression reduced the expression of SOX2 protein and vice versa. Knockdown of *SOX17* led to increased expression of SOX2, while enhanced expression of miR-371-5p abolished *SOX17* reponsiveness (Figure 4E).

**Figure 5:**
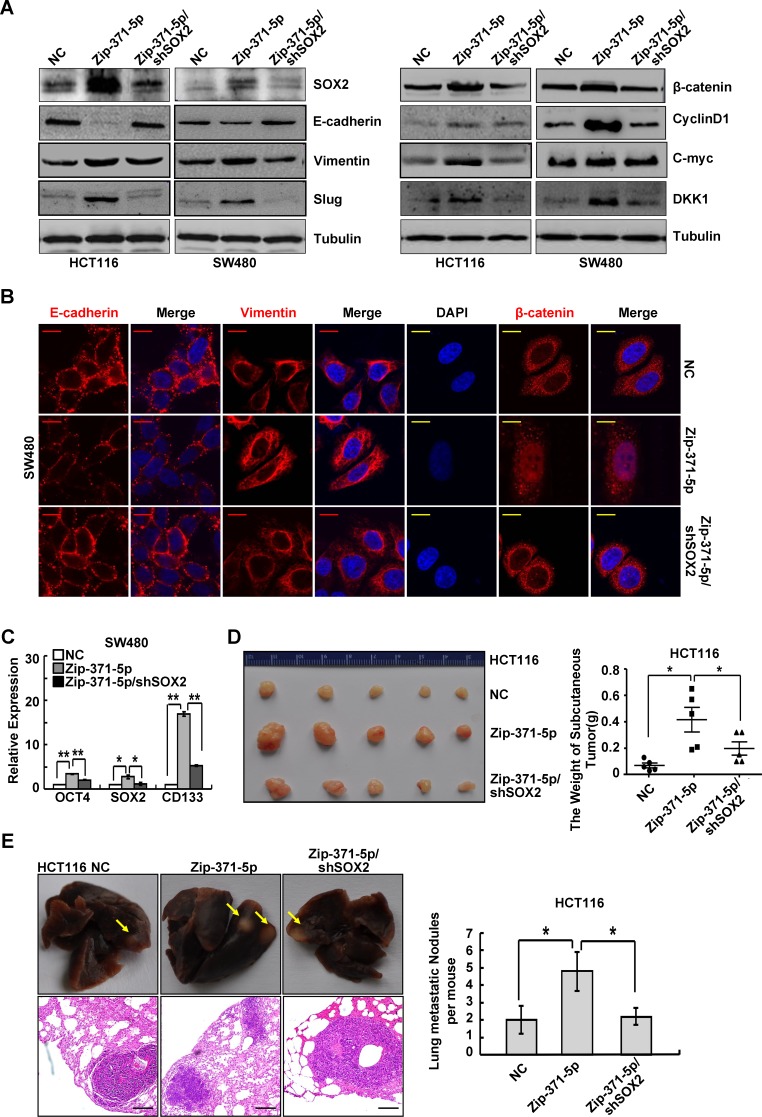
SOX2 is a functional target of miR-371-5p (A) Expression of EMT related markers and target genes of Wnt/β-catenin signaling in cells treated with Zip-371-5p or Zip-371-5p/shSOX2 by Western blot. Expression levels were normalized to Tubulin. (B) Immunofluorescence images of E-cadherin, Vimentin expression and nuclear translocation of β-catenin in SW480/NC, SW480/Zip-371-5p and SW480/Zip-371-5p/shSOX2 cells. Red scale bars represent 10 μm, whereas yellow scale bars represent 5 μm. (C) Expression of OCT4, SOX2 and CD133 in SW480 cells treated with Zip-371-5p or Zip-371-5p/shSOX2 by qRT-PCR. The relative expression levels in NC cells were normalized to 1. (D) HCT116/NC, HCT116/Zip-371-5p and HCT116/Zip-371-5p/shSOX2 cells (1×10^6^) were injected in the subcutaneous tissue of nude mice (n = 5). The weight of subcutaneous tumors was measured. (E) HCT116/NC, HCT116/Zip-371-5p and HCT116/Zip-371-5p/shSOX2 cells (2×10^6^) were injected into the tail vein of nude mice (n = 5) for 2 months. Yellow arrows in top panels point at lung metastatic nodules. Scale bars in bottom panels represent 100 μm. The number of lung metastatic nodules per mouse was counted under the microscope. * P < 0.05, ** P < 0.01. Data represent the mean ± SD.

Transcriptional regulator *SOX2* was identified as an oncogene in many cancers, including colon cancer. It plays an important role in cancer stem cell, EMT and metastasis of colon cancer [21-23]. To determine whether cancer cell phenotypes associated with miR-371-5p expression could be reversed via restoration of *SOX2*, we transfected miR-371-5p-depleting cells with shRNAs toward *SOX2* in HCT116 and SW480 cells (Supplementary Figure 5B). In miR-371-5p-depleting cells, depletion of *SOX2* reversed, at least partially, miR-371-5p knockdown-imposed proliferation and invasion (Supplementary Figure 5C). *SOX2* rescued miR-371-5p's dependent MET morphogical changes (Supplementary Figure 5D). Reintroduction of *SOX2* shRNAs in miR-371-5p depleting cells led to down-regulations of Vimentin and Slug, up-regulation of E-cadherin (Figure 5A and 5B), cytoplasmic translocation of β-catenin (Figure 5B), TCF/LEF transcriptional inactivation (Supplementary Figure 5E) and decreased expression of target genes CyclinD1, C-myc and DKK1 (Figure 5A). Moreover, depletion of *SOX2* abolished the promoting effect of miR-371-5p knockdown on stemness, as shown by decreased expression of stem cell pluripotency factors *OCT4*, *SOX2* and stem cell marker *CD133* (Figure 5C) and decreased sphere-forming capacity (Supplementary Figure 5F). We also evaluated whether *SOX2* rescued miR-371-5p's effects on tumor growth and metastasis *in vivo*. Depletion of *SOX2* reversed, at least in part, tumor growth and lung metastases resulting from knockdown of miR-371-5p (Figure 5D and 5E). These results reveal that miR-371-5p is a crucial and unexpected switch for EMT, stemness and metastasis of CRC via repression of *SOX2*.

### Correlations of miR-371-5p with SOX17, SOX2 expression in CRC cell lines and tissues

**Figure 6:**
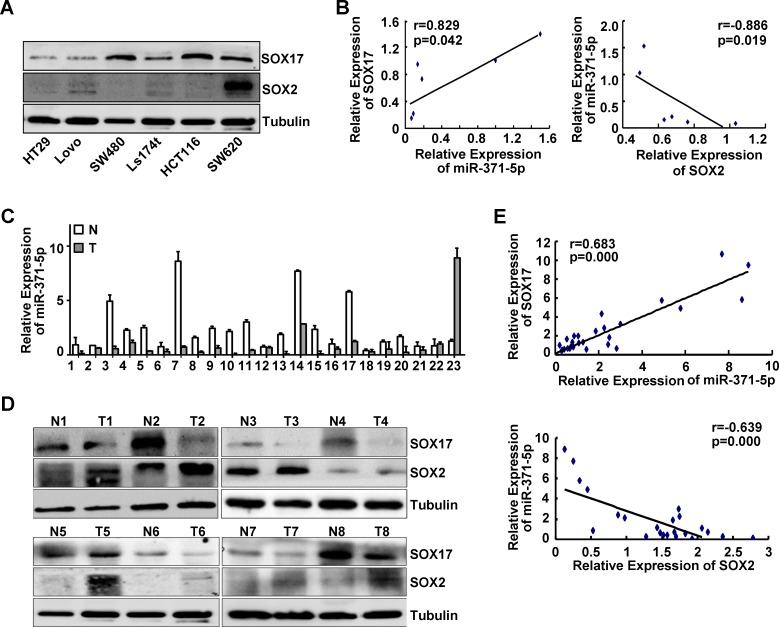
Expression correlations of miR-371-5p with SOX17, SOX2 in CRC cell lines and tissues (A) Endogenous expression of SOX17 and SOX2 in 6 CRC cell lines by Western blot. Expression levels were normalized to Tubulin. (B) Spearman's correlation between SOX17 and miR-371-5p expression levels, miR-371-5p and SOX2 expression levels in 6 CRC cell lines. Image density of SOX17 and SOX2 protein expression was determined by Gel densitometry. (C) Expression of miR-371-5p in 23 primary CRC tissues (T) and matched adjacent normal mucosa (N) by qRT-PCR. (D) Expression of SOX17 and SOX2 in 8 primary CRC tissues (T) and matched adjacent normal mucosa (N) by Western blot. Expression levels were normalized to Tubulin. (E) Spearman's correlation between SOX17 and miR-371-5p expression levels, miR-371-5p and SOX2 expression levels in 23 paired CRC specimens. Image density of SOX17 and SOX2 protein expression was determined by Gel densitometry.

To ascertain whether SOX17 could functionally affect miR-371-5p and SOX2 expression, we detected the expression levels of SOX17, miR-371-5p and SOX2 in a series of CRC cell lines and a matched collection of 23 human CRC tissues. Across the six cell lines tested we detected a significant positive correlation between SOX17 and miR-371-5p expression levels (*r* = 0.829, *p* < 0.05; Figure 1A, Figure 6A and 6B), and a negative one between miR-371-5p and SOX2 expression levels (*r* = −0.886, *p* < 0.05; Figure 1A, Figure 6A and 6B). In addition, miR-371-5p and SOX17 were obviously down-regulated, while SOX2 was markedly up-regulated in primary CRC tissues compared with adjacent normal mucosa (Figure 6C and 6D). There was a positive relationship between SOX17 and miR-371-5p expression levels (*r* = 0.683, *p* < 0.001; Figure 6E). Spearman's correlation analysis also showed a negative relationship between miR-371-5p and SOX2 expression levels (*r* = −0.639, *p* < 0.001; Figure 6E). These data verify that SOX17 induces miR-371-5p expression and consequently suppresses its direct target SOX2. The decreased expression of miR-371-5p can be one of the causes of high expression of SOX2 in CRC tissues.

## DISCUSSION

In this study, we first identify miR-371-5p as an oncosuppressor in CRC. We evaluated its expression in CRC cell lines and 100 paired cases of human primary CRC tissues. The findings showed that lower expression levels of miR-371-5p were seen in primary CRC tissues and miR-371-5p correlated significantly with differentiation, tumor size and metastasis. Recently, the altered expression of miR-371-5p has been reported in several tumors by using miRNA assay [12-15], but need to be further validated. Only one paper has reported miR-371-5p's roles in cancer. MiR-371-5p was up-regulated in hepatocellular cancer and promoted tumor growth by targeting *PRPF4B* [16]. Thus, the function of miR-371-5p in the progression of tumor remains unclear. We performed gain-of-function and loss-of-function assays to investigate the effect of miR-371-5p in the progression of CRC. MiR-371-5p could attenuate proliferation of CRC cells *in vitro*. This finding has yielded a contradiction to the recent study on the promoting role of miR-371-5p in HCC cell growth [16], which may be due to cancer heterogeneity. MiR-371-5p also suppressed EMT in CRC cells by regulating Wnt/β-catenin signaling, which was supported by data showing that miR-371-373 expression was correlated with Wnt/β-catenin signaling activity in several human cancer cell lines [24]. EMT confers mesenchymal properties on epithelial cells and has been closely associated with the acquisition of aggressive traits in cancer cells [25]. Indeed, we found that miR-371-5p knockdown induced invasiveness of CRC cells *in vitro*, and facilitated intestinal and hepatic metastases of CRC cells in orthotopic metastatic mouse model and lung colonization in tail vein metastatic mouse model. Recently, molecular links between EMT transcription factors and self-renewal have emerged, suggesting that EMT programs and CSCs play critical roles in both early and late stages of metastatic cascade [4, 25], but the linkage mechanism has not been fully elucidated. The microRNA-371-373 cluster was originally found to be specifically expressed in human embryonic stem cells [26] and has been involved in stem cell pluripotency [18, 19]. Our results also demonstrated that miR-371-5p strongly decreased the CRC stemness phenotypes. Thus, our data provide evidence that miR-371-5p is an important oncosuppressor in CRC growth, invasion, EMT, stemness and metastasis.

Having established miR-371-5p as an anti-metastatic miRNA in CRC, we next investigated possible mechanism of miR-371-5p in the progression of CRC. We performed a bioinformatics search for potential upstream transcription factors or target genes of miR-371-5p. The results showed that demethylation of transcription factor *SOX17* induced miR-371-5p expression and consequently suppressed its direct target *SOX2* in CRC cells. The SOX family is important for the maintenance of stem cells in multiple human tissues and tumors [27]. Recent studies indicate that several SOX family members such as *SOX2*, *SOX4* and *SOX18*, play vital roles in tumorigenesis and metastasis [28-30]. *SOX17* is epigenetically inactivated by promoter methylation in many cancers including colon cancer and regarded as a canonical Wnt antagonist [20, 31-33]. *SOX17* regulates proliferation, cell cycle and angiogenesis during cancer progression [34-36]. However, the association between *SOX17* and EMT, metastasis is unknown. Our results showed that *SOX17* was sufficient to suppress CRC cell proliferation, stemness and EMT by regulating β-catenin/TCF activity. Moreover, it inhibited invasion and metastasis in CRC cells. MiR-371-5p was necessary for *SOX17* mediated cancer-related traits in CRC cells. Thus, miR-371-5p is a functional target regulated by the transcription factor *SOX17*. *SOX2* plays multiple roles in stem cell maintenance and tumorigenesis [28]. *SOX2* is over-expressed in CRC tissues and regulates cancer cell growth *in vitro* and *in vivo* [23]. *SOX2* is also involved in the EMT process and predicts liver and lymph node metastasis of CRC patients [21]. In our study, we identified that *SOX2* was a novel target of miR-371-5p. MiR-371-5p reduced the expression of SOX2 *in vitro*. “*SOX2* rescue” experiments proved that miR-371-5p regulated proliferation, EMT, stemness, invasion and metastasis in CRC cells mainly by targeting *SOX2*. *SOX2* improves metastasis of breast and prostate cancer cells by promoting EMT through Wnt/β-catenin signal network. SOX2 can bind and activate the promoter region of β-catenin [37]. Our findings also demonstrated that *SOX2* was required for miR-371-5p-induced MET by regulating β-catenin/TCF activity in CRC cells. Hence, *SOX2* seems to be a major downstream effector of miR-371-5p in its target network in CRC cells.

After demonstrating the role of SOX17/miR-371-5p/SOX2 axis in the progression of CRC, we finally detected the expression correlations of SOX17, miR-371-5p and SOX2 in CRC cell lines and 23 paired cases of human primary CRC tissues. There were a positive relationship between SOX17 and miR-371-5p expression levels, and a negative one between miR-371-5p and SOX2 expression levels. This evidence clearly validates that *SOX17* induces miR-371-5p expression and consequently suppresses its direct target *SOX2* in CRC cells and tissues.

To conclude, our work sheds light on a poorly understood cascade of events involved in EMT, stem cell properties and metastasis in CRC cells. We identified miR-371-5p as an important oncosuppressor in the progression of CRC. Importantly, there was an intricate multistep cascade involved in this process: down-regulation of *SOX17* in CRC cells due to promoter hypermethylation induced the low expression level of miR-371-5p, which suppressed *SOX2* leading to activation of Wnt/β-catenin signaling. This in turn led to up-regulations of CyclinD1, C-myc and DKK1 and increased invasiveness, EMT, stemness and metastasis. We propose that interruption of the SOX17/miR-371-5p/SOX2 pathway may present a useful therapeutic approach for controlling CRC proliferation, invasion and metastasis.

## MATERIALS AND METHODS

### Cell lines, human tissue samples and animals

Human CRC cell lines Lovo, SW620, HT29, SW480, HCT116, LS174T and human embryonal kidney 293 cells were purchased from Shanghai Cell Bank of Type Culture Collection. The cell lines were freshly authenticated in last year. The cell lines were cultured in DMEM medium (GIBCO, Gaithersburg, MD, USA) supplemented with 10% fetal bovine serum (HyClone, Logan, USA) in 5% CO_2_ at 37°C. Images of CRC cells were taken by Olympus inverted microscope and were outputted by CellSens Dimension software. Paired tissues from primary CRC tissues and adjacent normal mucosa were collected from 100 patients who underwent CRC resection without prior radiotherapy and chemotherapy in Nanfang Hospital in 2008. These samples were snap-frozen in liquid nitrogen immediately after resection, and then stored at −8°C until needed. Four-to-six-week-old male athymic BALB/c-nu/nu mice were purchased from the Central Laboratory of Animal Science of Southern Medical University (Guangzhou, China), and maintained in a specific Pathogen Free environment. All protocols for animal studies were reviewed and approved by the Institutional Animal Care and Use Committee of Southern Medical University.

### Vectors construction and retroviral infection

Lentiviral constructs expressing miR-371-5p (Lenti-miR microRNA precursor clone collection; System Biosciences) or repressing miR-371-5p (miRZips lentiviral-based microRNA inhibition; System Biosciences) were packaged using the pPACKH1 lentivector Packaging Kit (System Biosciences). shRNAs towards SOX17, SOX2 (System Biosciences) were cloned into pSuper-retro-puro. Lentiviral constructs were used to infect CRC cells to establish cells stably expressing miR-371-5p or repressing miR-371-5p, SOX17 and SOX2. In the rescue experiments, SOX17-depleting cells were transfected with miR-371-5p vector and miR-371-5p-depleting cells were transfected with shRNAs towards SOX2.

### Quantitative real-time pCR

Total RNA was extracted from the tissues and cultured cells using Trizol reagent (Invitrogen, USA). The detection of miR-371-5p, miR-371-3p and RNU6B was performed with the All-in-One miRNA qRT-PCR Detection Kit (GeneCopeia). First-strand cDNA was synthesized using the PrimeScript RT Reagent Kit (Perfect Real Time, Takara) and amplified for the detection of *SOX17*, *SOX2*, *OCT4*, *CD133* and *GAPDH* using primers listed in Supplementary Table 2. The relative gene expression levels were calculated using the 2-comparative Ct (2^−ΔΔCt^) method. miRNA expression levels were normalized for RNU6B, whereas mRNA expression levels were normalized for *GAPDH*.

### Proliferation, plate colony formation, cell invasion assays *in vitro*

The proliferation, plate colony formation and invasion of transfected CRC cells were performed according to established protocols [38].

### Self-renewal assay

For self-renewal assay, cells were cultured and suspended in serum-free DMEM/F12 medium (Hyclone) supplemented with basic fibroblast growth factor (20ng/mL), EGF (20ng/mL), leukemia-inhibitory factor (10ng/mL; Invitrogen), and insulin (25 mg/mL; Sigma). Cells were seeded in 96-well plates at one cell per well dilutions. Primary CRC spheres were dissociated, counted and seeded again. The number of secondary spheres was counted after 2 weeks [39].

### Animal models

For *in vivo* tumor growth assay, xenograft tumors were generated for a month by subcutaneous injection of 1×10^6^ cells [38]. The weight of subcutaneous tumors was measured. For orthotropic metastasis assay, nude mice were anesthetized and their cecum was exteriorized by laparotomy. Subcutaneous tumors in different groups were cut into same size and were embedded into the mesentery at the tail end of cecum. The gut was reposited to the abdominal cavity prior to surgical sutures. The mice were sacrificed and all organs were removed for biopsy after six weeks. For tail vein metastasis assay, a total of 2×10^6^ cells were injected into the tail vein of nude mice. After 2 months, mice were sacrificed, and lung tissues were dissected and subjected to histological examination. Metastatic tumors were detected by H&E staining and quantified by counting metastatic lesions in each section. Images were taken by Olympus DP72 upright microscope and were outputted by DP2-BSW software.

### Luciferase activity assay

For 3′UTR luciferase reporter assays, the 3′UTR segments of SOX2, BTG3 and SOCS5 containing putative miR-371-5p binding site were amplified by PCR and inserted into the psiCHECK2 vector. A mutant construct specific for putative miR-371-5p binding site in SOX2 3′UTR was also generated using Quick Change Site-Directed Mutagenesis Kit (Angilent). The luciferase vectors and miR-371-5p-expressing vector were cotransfected by using Lipofectamine 2000 (Invitrogen). For the binding of SOX17 to miR-371-5p promoter, the coding region of SOX17 and the 1kb region directly upstream of miR-371-5p were amplified by PCR and then inserted into the vectors. The pGL3-miR-371-5p-Luc reporter was cotransfected with pRL-TK and either pcDNA-eGFP (control) or pcDNA-SOX17 into cells. Luciferase activity was measured 48 hours after transfection by the Dual-Luciferase Reporter Assay Kit (Promega). Primers are provided in Supplementary Table 3.

### Chromatin immunoprecipitation assay (ChIP)

For the ChIP Assay, cells were lysed using SDS lysis buffer and DNA was sheared optimized for about 500bp fragments by sonication. Protein-DNA complexes were precipitated by anti-SOX17 (Abcam) or anti-IgG antibody, then recovered using protein G agarose beads, washed, and eluted. Crosslinks were then reversed at 65°C overnight. The immunoprecipitated DNA was amplified by PCR for specific sequences (R1, R2 and R3) containing putative SOX17 binding sites. Primers are listed in Supplementary Table 3.

### Immunofluorescence and immunoblot analyses

Cells were cultured at a density of 1.5 × 10^5^ cells/well on 8 mm coverslips in 12-well plates. After 48 hours, coverslips were fixed by ice-cold methanol, and incubated with primary E-cadherin (Abcam), Vimentin and β-catenin (Epitomics) antibodies prior to florescent-labeled secondary antibodies. Nuclear DNA was stained with 4′, 6-diamidino-2-phenylindole (DAPI) and coverslips were mounted with FluorSave reagent (CALBIOCHEM). Immunofluorescence images were taken by Olympus inverted fluorescence microscope and were outputted by PV10-ASW 1.7 viewer software. Immunoblotting was performed as follows: Proteins were extracted with lysis buffer and then quantified by the BCA method (KeyGen Biotech). Lysates were diluted in SDS sample buffer (KeyGen Biotech) prior to SDS-PAGE, and then transferred to a polyvinylidene difluoride membrane (Roche Applied Sciences). Membranes were immunoblotted overnight at 4°C with anti-SOX17 (Millipore), anti-CyclinD1 (Abclonal), anti-C-myc, anti-DKK1 (Cell Signaling Technology), anti-E-cadherin (Abcam), anti-SOX2, anti-β-catenin, anti-Vimentin and anti-Slug and anti-N-cadherin antibodies (Epitomics), followed by the appropriate second antibodies. The bands were exposed using Pierce ECL Western Blotting Substrate (Thermo Scientific). Gel densitometry (Bio-Rad) was used to quantify immunoblot signals on exposed film.

### Statistical analysis

Quantitative values of all experiments are presented as the mean ± SD, which are calculated from 3 independent experiments. All statistical analyses were performed by SPSS 13.0 statistical software. Statistical significance among/between groups was tested using one-way ANOVA or the independent samples t-test. Relationships between miR-371-5p expression and clinicopathologic characteristics were performed with Fisher exact test. Pearson's or Spearman's correlation coefficient was used to measure the degree of the linear relationship of gene expression levels. *P* < 0.05 was considered to be statistically significant.

## SUPPLEMENTARY MATERIALS FIGURES AND TABLES


