# Supplementary material for: The SOX17/miR-371-5p/SOX2 axis inhibits EMT, stem cell properties and metastasis in colorectal cancer
Source: Oncotarget. 2015 Mar 15;6(11):9099–112. doi: 10.18632/oncotarget.3603 (PMC4496205; doi:10.18632/oncotarget.3603)
Supplement: Supplementary file 1 [file oncotarget-06-9099-s001.pdf]

The SOX17/miR-371-5p/SOX2 axis inhibits EMT, stem cell properties and metastasis in colorectal cancer

Supplementary Material

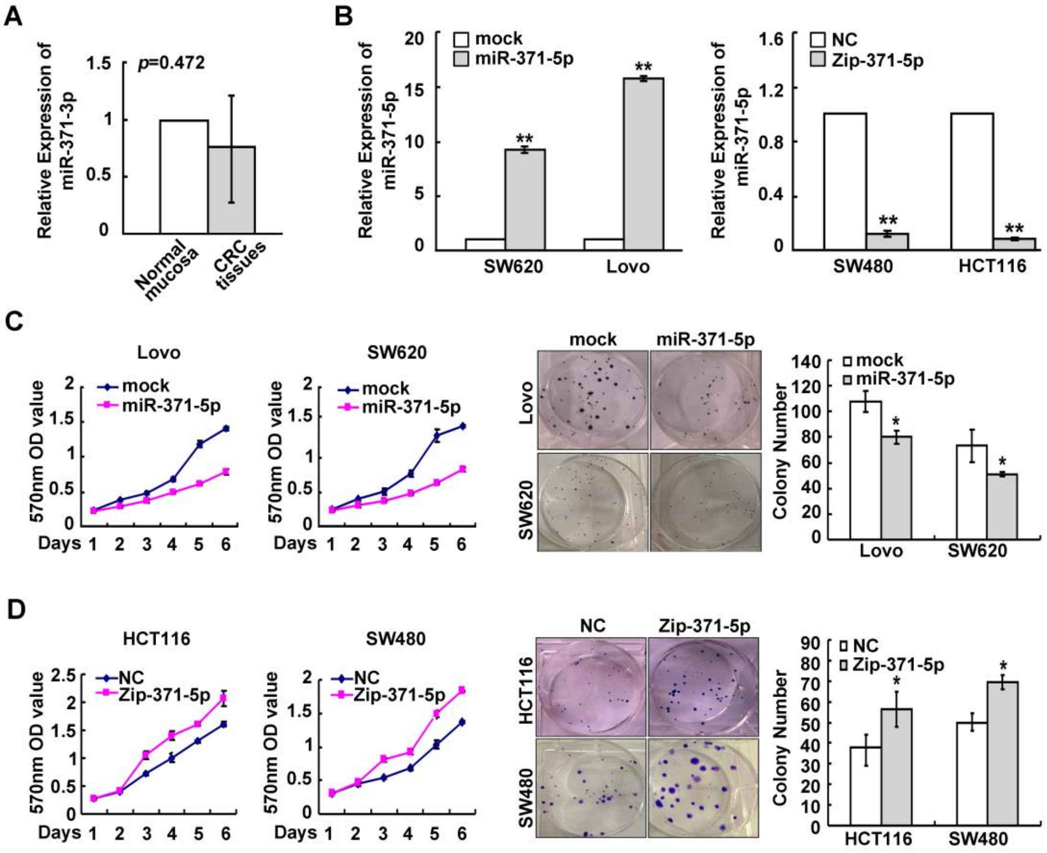

Supplementary Figure 1: miR-371-5p suppresses proliferation of CRC cells. (A) miR-371-3p expression in 100 cases of primary CRC tissues and matched adjacent normal mucosa by qRT-PCR. The relative expression levels of miR-371-3p in normal mucosa were normalized to 1. (B) miR-371-5p expression in cells transduced lentiviral vector expressing miR-371-5p or repressing miR-371-5p by qRT-PCR. The relative expression levels of miR-371-5p in mock or NC cells were normalized to 1. (C-D) Effect of miR-371-5p ectopic expression (C) or knockdown (D) on the proliferation of CRC cells by MTT assay and colony formation assay. * $P < 0.05$, ** $P < 0.01$. Data represent the mean ± SD.

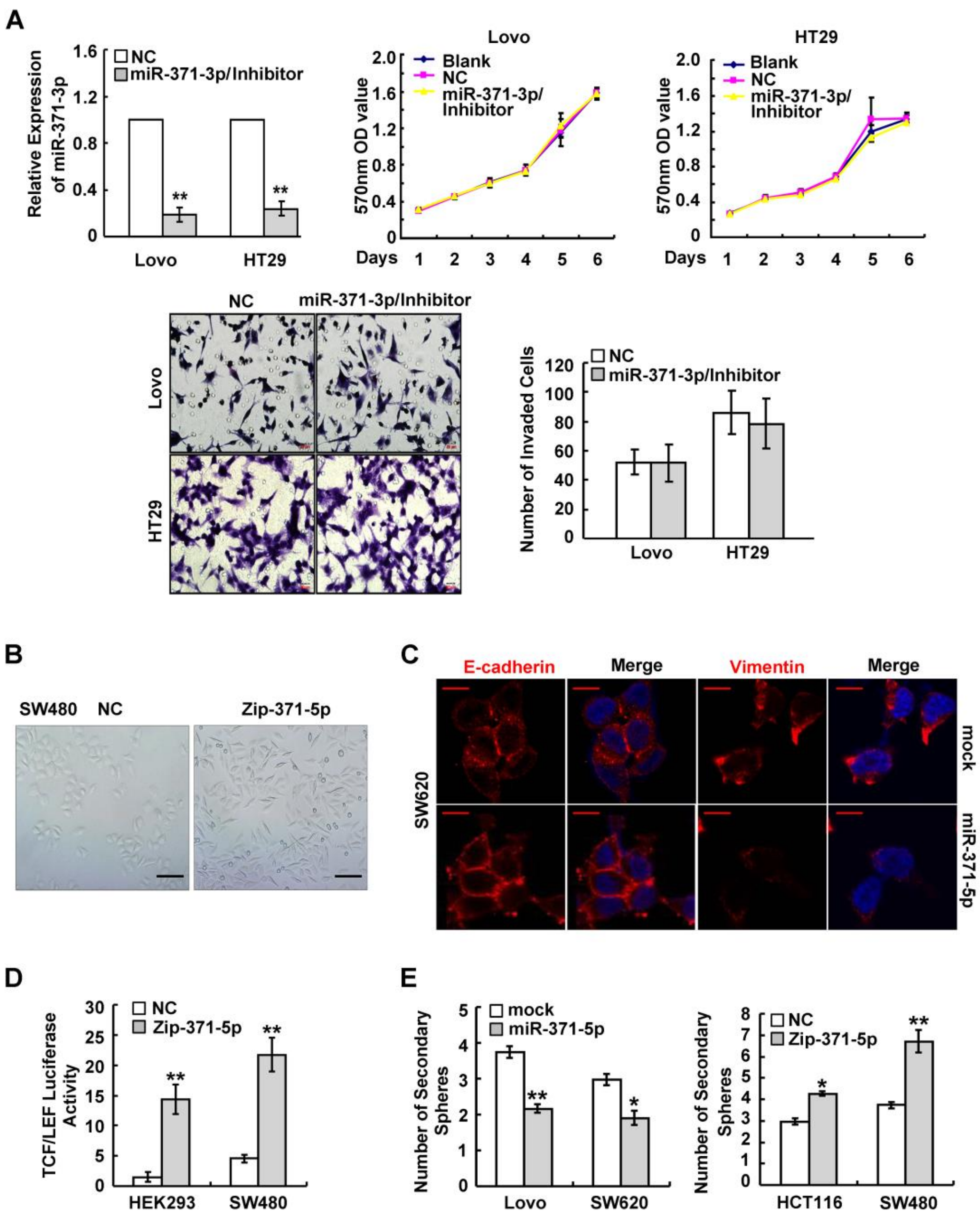

Supplementary Figure 2: miR-371-5p suppresses EMT and stem cell properties in CRC. (A) Effect of miR-371-3p inhibitor on the proliferation and invasiveness of CRC cells by MTT assay or Boyden chamber. Scale bars represent 20 μm. (B) Representative images of morphological change in miR-371-5p depleting cells. Scale bars represent 50 μm. (C) Immunofluorescence images of E-cadherin， Vimentin expression in miR-371-5p over-expressing cells. Red scale bars represent 10 μm. (D)

Luciferase activities of TCF/LEF transcription in HEK293 and SW480 cells treated with Zip-371-5p. (E) The number of secondary spheres from miR-371-5p over-expressing or depleting cells. * P < 0.05, ** P < 0.01. Data represent the mean ± SD.

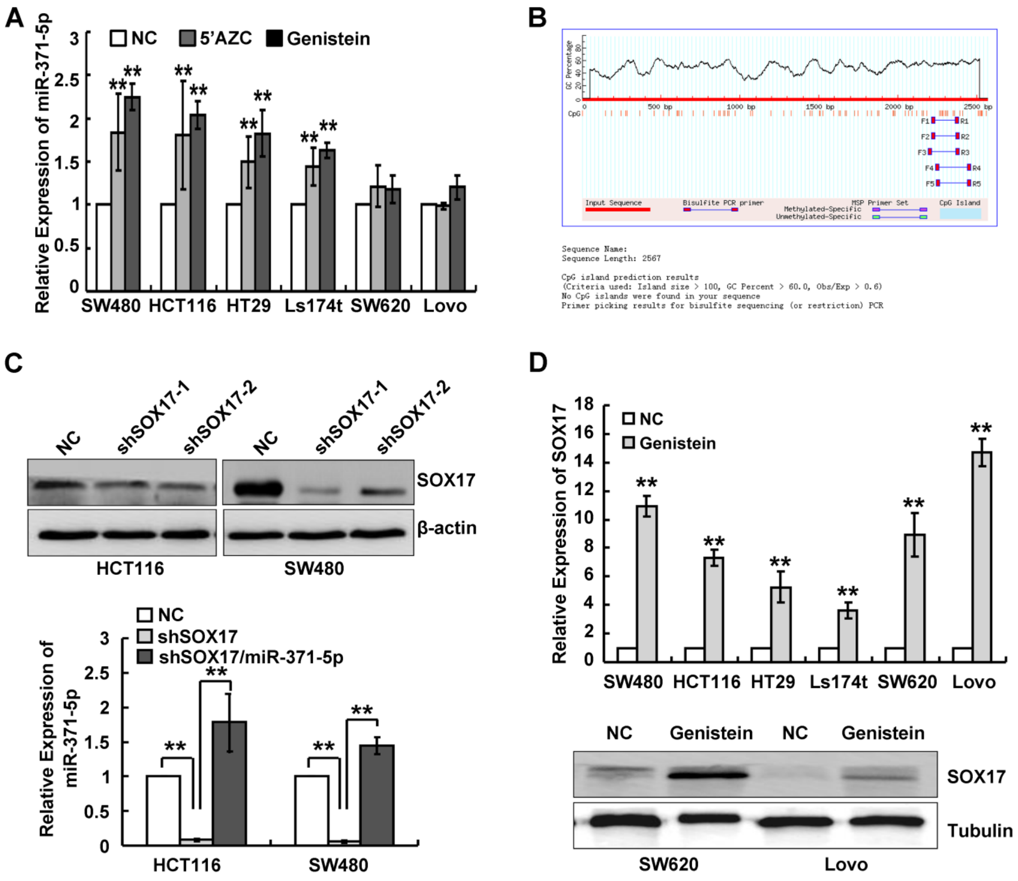

Supplementary Figure 3: Down-regulation of SOX17 is associated with promoter methylation. (A) miR-371-5p expression in 6 CRC cell lines treated with methyltransferase inhibitor 5'AZC or Genistein by qRT-PCR. The relative expression levels of miR-371-5p in CRC cells without treatment were normalized to 1. (B) Bioinformatic analysis of miR-371-5p promoter by Methyprimer software. (C) SOX17 expression in HCT116 and SW480 cells treated with shSOX17-1 or shSOX17-2 by Western blot. MiR-371-5p expression in HCT116 and SW480 cells

treated with shSOX17 or shSOX17/miR-371-5p by qRT-PCR. Protein expression levels were normalized to β-actin, while mRNA expression levels of miR-371-5p in NC cells were normalized to 1. (D) SOX17 expression in CRC cells treated with methyltransferase inhibitor Genistein by qRT-PCR or Western blot. mRNA expression levels of SOX17 in NC cells were normalized to 1， while protein expression levels were normalized to Tubulin. * $P < 0.05$, ** $P < 0.01$. Data represent the mean ± SD.

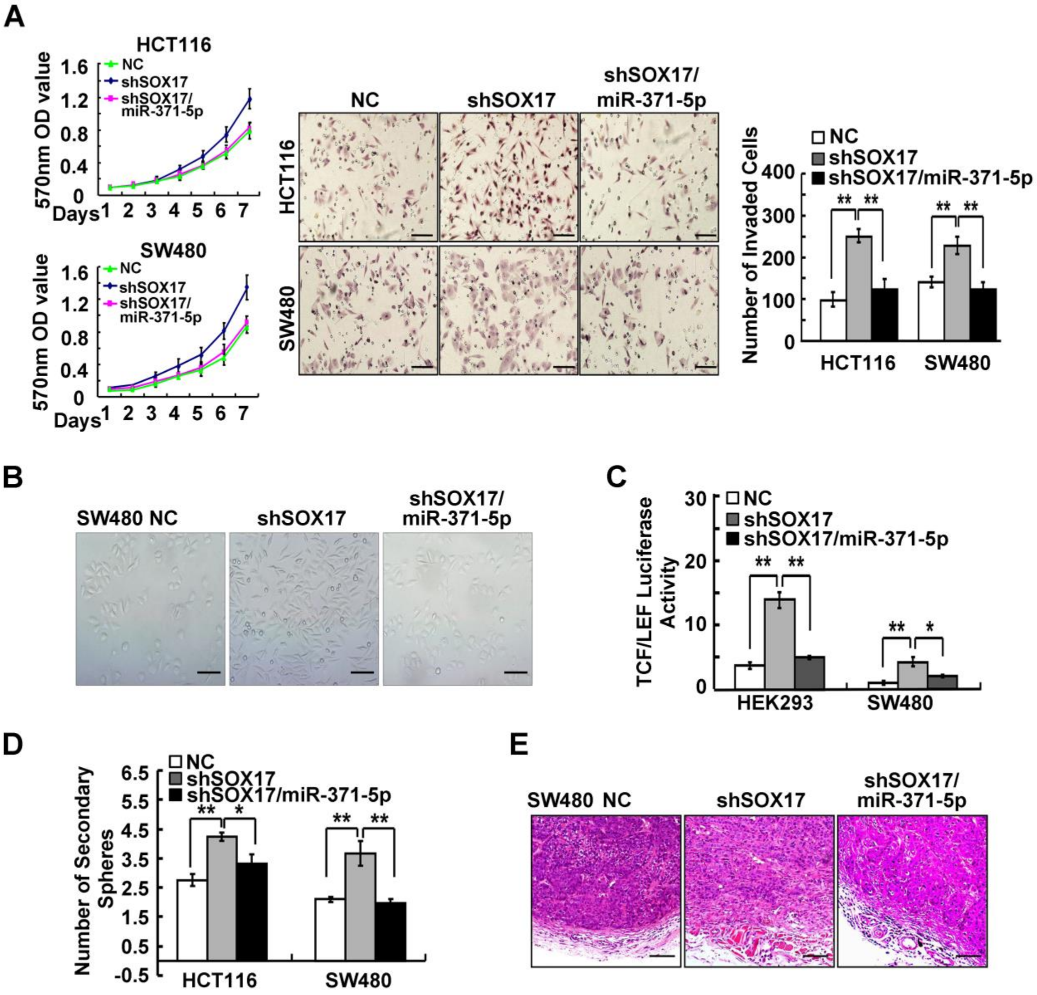

Supplementary Figure 4: SOX17 is sufficient to suppress cell proliferation, invasion, EMT and stem cell properties by targeting miR-371-5p in CRC. (A) Effects of shSOX17 and shSOX17/miR-371-5p on cell proliferation and invasiveness by MTT assay or Boyden chamber. Scale bars represent 50 μm. (B) Representative images of morphological change in SW480 cells treated with shSOX17 or shSOX17/miR-371-5p. Scale bars represent 50 μm. (C) Luciferase activities of TCF/LEF transcription in HEK293 and SW480 cells treated with shSOX17 or shSOX17/miR-371-5p. (D) The number of secondary spheres from cells treated with shSOX17 or shSOX17/miR-371-5p. (E) SW480/NC and SW480/shSOX17 and SW480/shSOX17/miR-371-5p cells (1×10^6) were injected in the subcutaneous tissue

of nude mice (n = 5). Local invasion of subcutaneous tumors by HE staining. Scale bars represent 50 μm. * P < 0.05, ** P < 0.01. Data represent the mean ± SD.

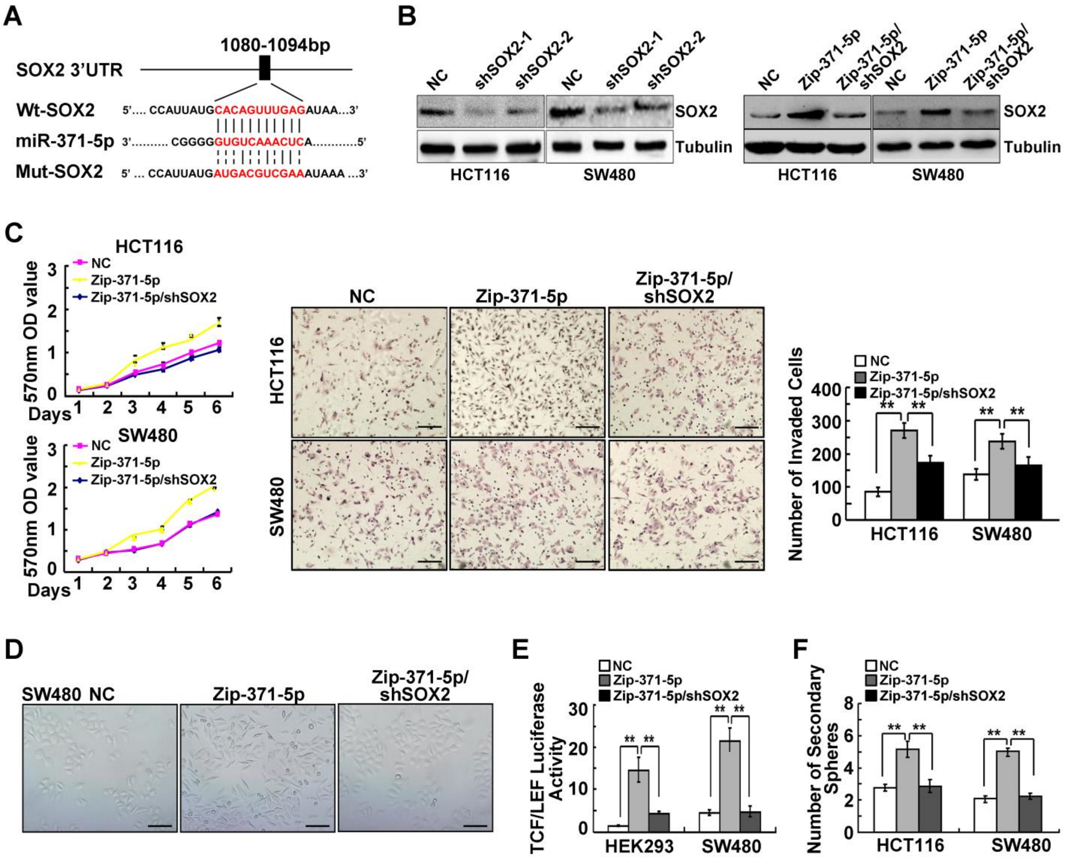

Supplementary Figure 5: miR-371-5p suppresses cell proliferation, invasion, EMT and stem cell properties by targeting SOX2 in CRC. (A) The sequence of mutated binding site of miR-371-5p in the SOX2 3′UTR.（B）SOX2 expression in HCT116 and SW480 cells treated with shSOX2-1, shSOX2-2, Zip-371-5p or Zip-371-5p/shSOX2 by Western blot. Expression levels were normalized to Tubulin. (C) Effects of Zip-371-5p and Zip-371-5p/shSOX2 on cell proliferation and invasiveness by MTT assay or Boyden chamber. Scale bars represent 100 μm. (D) Representative

images of morphological change in SW480/NC, SW480/Zip-371-5p and SW480/Zip-371-5p/shSOX2 cells. Scale bars represent 50 μm. (E) Luciferase activities of TCF/LEF transcription in HEK293 and SW480 cells treated with Zip-371-5p or Zip-371-5p/shSOX2. (F) The number of secondary spheres from cells treated with Zip-371-5p or Zip-371-5p/shSOX2. * $P < 0.05$, ** $P < 0.01$. Data represent the mean ± SD.

Supplementary Table 1: Relationship between miR-371-5p expression and clinicopathologic features of CRC patients.

**Features**	**Number**	$2^{-\triangle\triangle Ct}$ **（mean±SD）**	**F/t**	***p***
**All case**	100			
**Age (years)** [a]				
<50	28	0.268±0.206	t=-1.324	*p*=0.189
≥50	72	0.345±0.161		
**Gender**				
Male	55	0.361±0.208	t=0.558	*p*=0.557
Female	44	0.302±0.132		
**Position**				
Colon	47	0.342±0.197	t=0.622	*p*=0.535
Rectal	53	0.304±0.155		
**Differentiation**				
Well	19	0.372±0.270		
Moderate	57	0.338±0.158	**F=5.780**	***p*=0.004**
Poor	24	0.239±0.080		
**Infiltration Depth**				
Muscular layer	18	0.409±0.375		
Full-thickness	55	0.299±0.104	F=1.996	*p*=0.141
Serosa	27	0.307±0.226		
**Tumor Size (cm)**[b]				
<5cm	57	0.393±0.268	**t=-2.814**	***p*=0.006**
≥5cm	43	0.216±0.103		
**Lymphatic Metastasis**				
N	36	0.464±0.151	**t=2.260**	***p*=0.037**
Y	64	0.285±0.219		
**Liver Metastasis**				
N	85	0.347±0.252	**t=2.098**	***p*=0.043**
Y	15	0.184±0.122		

[a] Age was grouped according to established rules.

[b] Tumor size was grouped according to median.

Supplementary Table 2: Primer sequences for Quantitative Real-Time PCR.

Gene	Primers	Sequence(5’-3’)
SOX2	Forward	TACAGCATGTCCTACTCGCAG
	Reverse	GAGGAAGAGGTAACCACAGGG
OCT4	Forward	GTGTTCAGCCAAAAGACCATCT
	Reverse	GGCCTGCATGAGGGTTTCT
SOX17	Forward	GTGGACCGCACGGAATTTG
	Reverse	GGAGATTCACACCGGAGTCA
CD133	Forward	AGTCGGAAACTGGCAGATAGC
	Reverse	GGTAGTGTTGTACTGGGCCAAT
GAPDH	Forward	TGTGGGCATCAATGGATTTGG
	Reverse	ACACCATGTATTCCGGGTCAAT

Supplementary Table 3: Primer sequences for PCR amplification.

Gene	Primers	Sequence(5’-3’)
BTG3-3'UTR Wt	Forward	AAAACTCGAGTCGTTTTTGATTGTGTTGGTGTC
	Reverse	AAAAGCGGCCGCTGCCTCAAACTGTTTTTATTTGCA
SOX2-3'UTR Wt	Forward	AATACTCGAGAGCATGGAGAAAACCCGGTA
	Reverse	TAAAGCGGCCGCAATTTATTTATCTCAAACTGTGCA
SOCS5-3'UTR Wt	Forward	AAAACTCGAGCAATAGCGGATAGAGCTACAGGT
	Reverse	AAAAGCGGCCGCTAGTTGACAGGCTACAATGGGA
SOX2-3'UTR Mut	Forward	ATGACGTCGAAATAAATAAATAAATT
	Reverse	CATAATGGAGTAAAAACTTAAGTTGA
SOX17-CDS	Forward	GCGGTAGGCGTGTACGGT
	Reverse	CCGGACACGCTGAACTTGT
miR-371-5p Promoter	Forward	AATTGGTACCGTGGGCTCTCACCCTATATAAGAAC
	Reverse	TAGACTCGAGGACATCAGAATAGTGCTCCACATTT
R1(-996— -654)	Forward	GAGTGGATGACTGGTGGAATG
	Reverse	TCACAGAGGCCAATGATACG
R2（-777— -361）	Forward	GGAAGGGGTGGGCTTTAA
	Reverse	TCGGAAGGCTACGGTGGTA
R3（-376— -86）	Forward	CACCGTAGCCTTCCGAGTA
	Reverse	GCCCTTGATGAGCTGTTGA
